# Supplementary material for: Fused 1,2-Diboraoxazoles Based on closo-Decaborate Anion–Novel Members of Diboroheterocycle Class
Source: Molecules. 2021 Jan 5;26(1):248. doi: 10.3390/molecules26010248 (PMC7796516; doi:10.3390/molecules26010248)
Supplement: Supplementary file 1 [file molecules-26-00248-s001.pdf]

Supplementary Materials

Fused 1,2-diboraoxazoles based on closo-decaborate anion – novel members of diboroheterocycle class

Vera V. Voinova [1], Nikita A. Selivanov [1], Ivan V. Plyushchenko [2], Mikhail F. Vokuev [2], Alexander Yu. Bykov [1], Ilya N. Klyukin [1], Alexander S. Novikov [3], Andrey P. Zhdanov [1,*], Mikhail S. Grigoriev [4], Igor A. Rodin [2], Konstantin Yu. Zhizhin [1] and Nikolay T. Kuznetsov [1]

1 Kurnakov Institute of General and Inorganic Chemistry, Russian Academy of Sciences, 119991, Leninskii pr. 31, Moscow, Russian Federation; veravoinova@rx24.ru, GooVee@yandex.ru, bykov@igic.ras.ru, klukinil@gmail.com, zhizhin@igic.ras.ru, ntkuz@igic.ras.ru.

2 Lomonosov Moscow State University, Chemistry department, 119991. Moscow, Russian Federation; plyush1993@bk.ru, vokuevmihail11@gmail.com, igorrodin@yandex.ru

3 Saint Petersburg State University, Institute of Chemistry, 199034, Universitetskaya Nab. 7-9, Saint Petersburg, Russian Federation; ja2-88@mail.ru

4 Frumkin Institute of Physical Chemistry and Electrochemistry, Russian Academy of Sciences, 119071, Leninskii pr. 31, Bldg 4, Moscow, Russian Federation; mickgrig@mail.ru

* Correspondence: zhdanov@igic.ras.ru; Tel.: +7-926-727-0139 (A.P.)

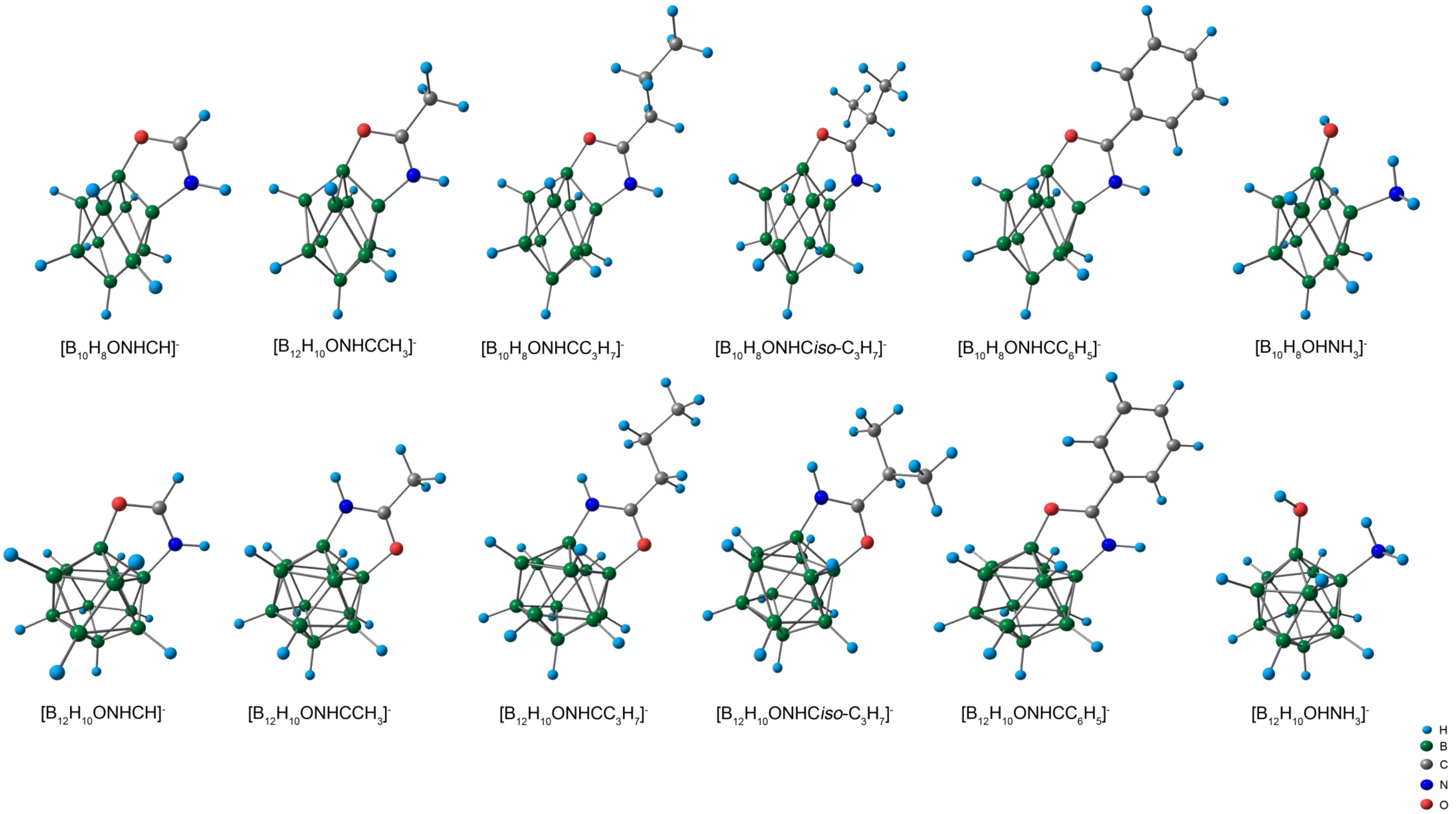

Figure S1. Optimized structures of *closo*-borate derivatives of general type $[B_nH_{n-2}ONHCR]^-$ and $[B_nH_{n-2}OHNH_3]^-$ (n = 10, 12; R = H, CH_3, C_3H_7, *iso*-C_3H_7, C_6H_5).

	Bond length (Å)	$\rho(r)$ (e Å^{-3})	$\nabla^2\rho(r)$ (e Å^{-5})	H_b (h e^{-1})	ε_b	δ(B–C)	Wiberg Index
$[B_{10}H_8ONCH]^-$							
B-O	1.51	0.131	0.658	-0.072	0.004	0.5236	0.64
B-N	1.54	0.141	0.539	-0.094	0.172	0.5564	0.73
C-O	1.28	0.349	-0.134	-0.575	0.071	1.0113	1.28
C-N	1.31	0.363	-0.903	-0.624	0.240	1.2045	1.49
$[B_{10}H_8ONCCH_3]^-$							
B-O	1.51	0.133	0.664	-0.073	0.007	0.5232	0.64
B-N	1.54	0.142	0.538	-0.096	0.172	0.5577	0.73
C-O	1.29	0.346	-0.194	-0.570	0.057	0.9791	1.26
C-N	1.31	0.361	-0.951	-0.618	0.231	1.1712	1.46
$[B_{10}H_8ONCC_3H_7]^-$							
B-O	1.51	0.133	0.667	-0.073	0.006	0.5233	0.642
B-N	1.54	0.143	0.538	-0.096	0.170	0.5582	0.7333
C-O	1.29	0.345	-0.180	-0.567	0.048	0.9741	1.25
C-N	1.31	0.361	-0.941	-0.617	0.224	1.1701	1.466
$[B_{10}H_8ONC\textit{iso}\text{-}C_3H_7]^-$							
B-O	1.51	0.133	0.670	-0.073	0.005	0.522	0.64
B-N	1.54	0.142	0.537	-0.096	0.174	0.5613	0.73
C-O	1.29	0.344	-0.150	-0.564	0.040	0.9682	1.25
C-N	1.31	0.361	-0.936	-0.617	0.225	1.1661	1.47
$[B_{10}H_8ONCC_6H_5]^-$							
B-O	1.50	0.133	0.679	-0.073	0.001	0.525	0.64
B-N	1.53	0.143	0.543	-0.096	0.167	0.560	0.73
C-O	1.29	0.345	-0.188	-0.566	0.061	0.973	1.25
C-N	1.31	0.360	-0.963	-0.614	0.233	1.163	1.45
$[B_{10}H_8OHNH_3]^-$							
B-O	1.45418	0.158	0.753	-0.093	0.037	0.572	0.78
B-N	1.58088	0.126	0.509	-0.078	0.217	0.542	0.69
$[B_{12}H_{10}ONHCH]^-$							
B-O	1.53	0.126	0.581	-0.072	0.013	0.499	0.63
B-N	1.54	0.141	0.510	-0.096	0.018	0.551	0.73
C-O	1.28	0.350	-0.144	-0.577	0.058	1.012	1.28
C-N	1.31	0.363	-0.885	-0.624	0.232	1.202	1.49
$[B_{12}H_{10}ONCCH_3]^-$							
B-O	1.53	0.128	0.595	-0.073	0.015	0.500	0.63
B-N	1.54	0.143	0.509	-0.099	0.020	0.553	0.73
C-O	1.29	0.347	-0.204	-0.571	0.043	0.978	1.26
C-N	1.31	0.361	-0.936	-0.618	0.222	1.166	1.46
$[B_{12}H_{10}ONCC_3H_7]^-$							
B-O	1.53	0.128	0.596	-0.073	0.016	0.500	0.63
B-N	1.54	0.143	0.509	-0.099	0.020	0.5529	0.73
C-O	1.29	0.346	-0.206	-0.569	0.036	0.974	1.26

C-N	1.31	0.361	-0.920	-0.617	0.212	1.163	1.46
$[B_{12}H_{10}ONC\textit{iso}\text{-}C_3H_7]^-$							
B-O	1.52	0.129	0.601	-0.073	0.015	0.5007	0.63
B-N	1.54	0.143	0.508	-0.099	0.019	0.5518	0.73
C-O	1.29	0.345	-0.193	-0.566	0.031	0.9701	1.25
C-N	1.31	0.360	-0.906	-0.616	0.208	1.1625	1.47
$[B_{12}H_{10}ONCC_6H_5]^-$							
B-O	1.52	0.128	0.603	-0.072	0.014	0.4988	0.63
B-N	1.54	0.143	0.512	-0.098	0.019	0.5533	0.73
C-O	1.29	0.346	-0.204	-0.569	0.046	0.9743	1.25
C-N	1.31	0.360	-0.952	-0.615	0.222	1.1624	1.45
$[B_{12}H_{10}OHNH_3]^-$							
B-O	1.46	0.158	0.708	-0.097	0.036	0.556	0.77
B-N	1.58	0.129	0.497	-0.082	0.025	0.540	0.70

Table S1. Bond lengths**,** Wiberg index, and main topological parameters of electron density for interactions in diboraoxazole rings of $[B_nH_{n-2}ONHCR]^-$ (n = 10, 12; R = H, CH_3, C_3H_7, *iso*-C_3H_7). $\rho(r)$ – electron density at the bcp, $\nabla^2\rho(r)$ – Laplacian of electron density at the bcp, H_b – total energy at the bcp. δ(B–C) – delocalization index, ε_b – ellipticity at the bcp.

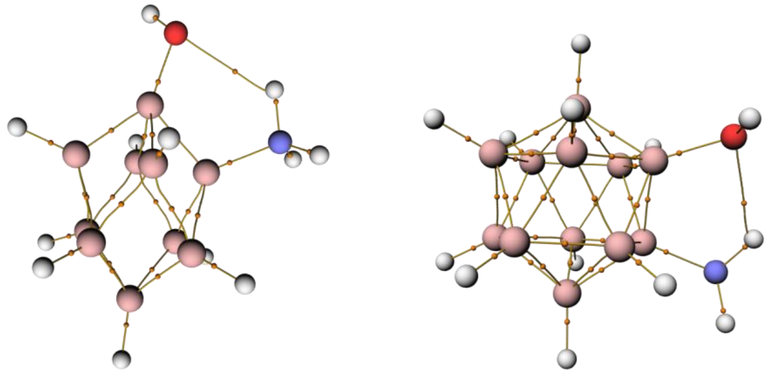

$[B_{10}H_8OHNH_3]^-$ $[B_{12}H_{10}OHNH_3]^-$

Figure S2. Molecular graphs for *closo*-borate derivatives of general type $[B_nH_{n-2}OHNH_3]^-$ (n = 10, 12). Bond critical points are indicated by small orange circles.

Bond length (Å)	$\rho(r)$ (e $Å^{-3}$)	$\nabla^2\rho(r)$ (e $Å^{-5}$)	H_b (h e^{-1})	ε_b	δ(B–C)	Wiberg Index
$[B_{10}H_8OHNH_3]^-$						
2.22	0.016	0.056	0.001	0.664	0.0508	0.0202
$[B_{12}H_{10}OHNH_3]^-$						
2.15509	0.018	0.059	0.000	0.321	0.057	0.024

Table S2. Bond lengths**,** Wiberg index, and main topological parameters of electron density for NH---O interactions in $[B_nH_{n-2}OHNH_3]^-$ (n = 10, 12). $\rho(r)$ – electron density at the bcp, $\nabla^2\rho(r)$ – Laplacian of electron density at the bcp, H_b – total energy at the bcp. δ(B–C) – delocalization index, ε_b – ellipticity at the bcp.

Compound	Atom	NBO	QTAIM

$[B_{10}H_8ONHCH]^-$			
	O	-1.32	-0.58
	N	-1.52	-0.71
	H	0.43	0.44
	$C_{carb.}$	1.53	0.52
	$B_{O\text{-}sub.}$	-0.08	0.22
	$B_{N\text{-}sub.}$	0.28	0.11
	B	0.63	-0.20
	H	-0.64	0.05
	B	0.75	-0.17
	H	-0.64	0.04
	B	0.63	-0.20
	H	-0.64	0.05
	B	0.60	-0.19
	H	-0.65	0.04
	B	0.60	-0.19
	H	-0.65	0.04
	B	0.56	-0.18
	H	-0.65	0.04
	B	0.56	-0.18
	H	-0.65	0.04
	B	0.43	-0.23
	H	-0.65	0.03
	H	0.05	0.20
$[B_{10}H_8ONCCH_3]^-$			
	O	-1.33	-0.59
	N	-1.52	-0.71
	H	0.43	0.44
	$C_{carb.}$	1.48	0.71

	C	0.06	-0.73
	H	0.03	0.26
	H	0.00	0.24
	$B_{O\text{-}sub.}$	-0.05	0.22
	$B_{N\text{-}sub.}$	0.29	0.12
	B	0.62	-0.20
	H	-0.64	0.04
	B	0.75	-0.17
	H	-0.65	0.04
	B	0.62	-0.20
	H	-0.64	0.04
	B	0.60	-0.19
	H	-0.65	0.04
	B	0.60	-0.19
	H	-0.65	0.04
	B	0.56	-0.18
	H	-0.65	0.04
	B	0.56	-0.18
	H	-0.65	0.04
	B	0.43	-0.24
	H	-0.65	0.03
	H	0.03	0.26
$[B_{10}H_8ONCC_3H_7]^-$			
	O	-1.33	-0.59
	N	-1.52	-0.71
	H	0.43	0.44
	$C_{carb.}$	1.46	0.72
	C	0.08	-0.52
	H	0.01	0.26

	H	-0.02	0.24
	C	0.09	-0.47
	H	-0.01	0.24
	H	0.01	0.26
	C	0.05	-0.68
	H	-0.02	0.24
	H	-0.03	0.22
	H	-0.02	0.23
	$B_{O\text{-}sub.}$	-0.05	0.22
	$B_{N\text{-}sub.}$	0.29	0.12
	B	0.62	-0.20
	H	-0.64	0.04
	B	0.74	-0.17
	H	-0.65	0.04
	B	0.62	-0.20
	H	-0.64	0.04
	B	0.60	-0.19
	H	-0.65	0.04
	B	0.60	-0.19
	H	-0.65	0.04
	B	0.56	-0.18
	H	-0.65	0.04
	B	0.56	-0.18
	H	-0.65	0.04
	B	0.43	-0.24
	H	-0.65	0.03
$[B_{10}H_8ONC\mathit{iso}\text{-}C_3H_7]^-$			
	O	-1.33	-0.60

	N	-1.52	-0.71
	H	0.43	0.44
	$C_{carb.}$	1.45	0.73
	C	0.10	-0.33
	H	-0.03	0.24
	C	0.05	-0.67
	H	0.00	0.24
	H	0.02	0.25
	H	-0.02	0.23
	C	0.05	-0.67
	H	0.00	0.24
	H	-0.03	0.23
	H	0.02	0.25
	$B_{O\text{-}sub.}$	-0.04	0.22
	$B_{N\text{-}sub.}$	0.28	0.11
	B	0.62	-0.20
	H	-0.64	0.04
	B	0.74	-0.17
	H	-0.65	0.04
	B	0.62	-0.20
	H	-0.64	0.04
	B	0.60	-0.19
	H	-0.65	0.04
	B	0.61	-0.19
	H	-0.65	0.04
	B	0.56	-0.18
	H	-0.65	0.04
	B	0.55	-0.18
	H	-0.65	0.04

	B	0.43	-0.24
	H	-0.65	0.03
$[B_{10}H_8ONCC_6H_5]^-$			
	O	-1.32	-0.58
	N	-1.51	-0.70
	H	0.43	0.44
	$C_{carb.}$	1.47	0.70
	$B_{O\text{-}sub.}$	-0.06	0.22
	$B_{N\text{-}sub.}$	0.28	0.12
	B	0.63	-0.20
	H	-0.64	0.05
	B	0.75	-0.17
	H	-0.65	0.04
	B	0.63	-0.20
	H	-0.64	0.05
	B	0.60	-0.19
	H	-0.65	0.04
	B	0.60	-0.19
	H	-0.65	0.04
	B	0.56	-0.18
	H	-0.65	0.04
	B	0.56	-0.18
	H	-0.65	0.04
	B	0.43	-0.23
	H	-0.65	0.03
	C	0.01	-0.12
	C	-0.01	-0.20
	C	0.01	-0.20
	C	0.00	-0.25

	C	0.00	-0.24
	C	-0.01	-0.23
	H	0.02	0.25
	H	0.06	0.27
	H	0.01	0.24
	H	0.01	0.25
	H	0.01	0.24
$[B_{10}H_8OHNH_3]^-$			
	O	-1.40	-0.96
	N	-1.31	-0.93
	H	0.45	0.45
	$B_{O\text{-}sub.}$	0.48	0.29
	$B_{N\text{-}sub.}$	0.03	0.06
	B	0.57	-0.22
	H	-0.64	0.05
	B	0.61	-0.20
	H	-0.64	0.04
	B	0.53	-0.23
	H	-0.65	0.04
	B	0.57	-0.20
	H	-0.66	0.03
	B	0.57	-0.20
	H	-0.66	0.03
	B	0.57	-0.17
	H	-0.65	0.04
	B	0.57	-0.18
	H	-0.65	0.04
	B	0.44	-0.24
	H	-0.65	0.03

	H	0.49	0.46
	H	0.45	0.45
	H	0.58	0.50
$[B_{12}H_{10}ONHCH]^{-}$			
	O	-1.31	-0.59
	N	-1.52	-0.71
	H	0.44	0.44
	$C_{carb.}$	1.54	0.54
	$B_{O\text{-}sub.}$	0.18	0.31
	$B_{N\text{-}sub.}$	0.23	0.12
	B	0.55	-0.21
	H	-0.64	0.05
	B	0.62	-0.19
	H	-0.64	0.06
	B	0.62	-0.19
	H	-0.64	0.06
	B	0.54	-0.21
	H	-0.63	0.05
	B	0.60	-0.18
	H	-0.64	0.05
	B	0.60	-0.18
	H	-0.64	0.05
	B	0.57	-0.18
	H	-0.64	0.06
	B	0.55	-0.18
	H	-0.64	0.06
	B	0.58	-0.17
	H	-0.64	0.05
	B	0.54	-0.19

	H	-0.64	0.06
	H	0.06	0.21
$[B_{12}H_{10}ONHCCH_3]^-$			
	O	-1.32	-0.60
	N	-1.52	-0.72
	H	0.43	0.44
	$C_{carb.}$	1.49	0.73
	$B_{O\text{-}sub.}$	0.20	0.31
	$B_{N\text{-}sub.}$	0.24	0.13
	B	0.54	-0.21
	H	-0.64	0.05
	B	0.61	-0.19
	H	-0.64	0.06
	B	0.62	-0.19
	H	-0.64	0.06
	B	0.53	-0.21
	H	-0.63	0.05
	B	0.60	-0.18
	H	-0.64	0.05
	B	0.60	-0.18
	H	-0.64	0.05
	B	0.57	-0.18
	H	-0.64	0.06
	B	0.55	-0.18
	H	-0.64	0.05
	B	0.57	-0.18
	H	-0.64	0.05
	B	0.54	-0.19
	H	-0.64	0.05

	C	0.06	-0.73
	H	0.00	0.24
	H	0.04	0.27
	H	0.04	0.27
$[B_{12}H_{10}ONHCC_3H_7]^-$			
	O	-1.32	-0.60
	N	-1.53	-0.72
	H	0.44	0.44
	$C_{carb.}$	1.48	0.74
	$B_{O\text{-}sub.}$	0.21	0.31
	$B_{N\text{-}sub.}$	0.24	0.13
	B	0.54	-0.21
	H	-0.64	0.05
	B	0.61	-0.19
	H	-0.64	0.06
	B	0.61	-0.19
	H	-0.64	0.06
	B	0.53	-0.21
	H	-0.63	0.05
	B	0.60	-0.18
	H	-0.64	0.05
	B	0.60	-0.17
	H	-0.65	0.05
	B	0.57	-0.18
	H	-0.64	0.05
	B	0.55	-0.18
	H	-0.64	0.05
	B	0.57	-0.17
	H	-0.64	0.05

	B	0.54	-0.19
	H	-0.64	0.05
	C	0.07	-0.53
	H	0.02	0.27
	H	0.02	0.27
	C	0.08	-0.48
	H	-0.02	0.24
	H	-0.02	0.24
	C	0.06	-0.67
	H	-0.02	0.23
	H	-0.02	0.23
	H	-0.02	0.23
$[B_{12}H_{10}ONHC\textit{iso}\text{-}C_3H_7]^-$			
	O	-1.32	-0.61
	N	-1.53	-0.72
	H	0.44	0.44
	$C_{carb.}$	1.47	0.74
	$B_{O\text{-}sub.}$	0.21	0.31
	$B_{N\text{-}sub.}$	0.25	0.13
	B	0.54	-0.20
	H	-0.64	0.05
	B	0.61	-0.19
	H	-0.64	0.06
	B	0.61	-0.19
	H	-0.64	0.06
	B	0.53	-0.21
	H	-0.63	0.05
	B	0.60	-0.18

	H	-0.64	0.05
	B	0.60	-0.18
	H	-0.64	0.05
	B	0.57	-0.18
	H	-0.64	0.06
	B	0.55	-0.18
	H	-0.64	0.05
	B	0.57	-0.18
	H	-0.64	0.05
	B	0.54	-0.18
	H	-0.64	0.06
	C	0.10	-0.34
	H	0.01	0.27
	C	0.04	-0.68
	H	-0.02	0.23
	H	-0.01	0.24
	H	0.00	0.24
	C	0.05	-0.67
	H	0.03	0.26
	H	-0.02	0.23
	H	0.00	0.24
$[B_{12}H_{10}ONHCC_6H_5]^-$			
	O	-1.31	-0.59
	N	-1.51	-0.71
	H	0.44	0.44
	$C_{carb.}$	1.48	0.72
	$B_{O\text{-}sub.}$	0.19	0.31
	$B_{N\text{-}sub.}$	0.24	0.13
	B	0.54	-0.20

	H	-0.64	0.05
	B	0.61	-0.19
	H	-0.64	0.06
	B	0.62	-0.19
	H	-0.64	0.06
	B	0.54	-0.21
	H	-0.63	0.05
	B	0.60	-0.18
	H	-0.64	0.05
	B	0.60	-0.18
	H	-0.64	0.05
	B	0.57	-0.18
	H	-0.64	0.06
	B	0.55	-0.18
	H	-0.64	0.06
	B	0.57	-0.17
	H	-0.64	0.05
	B	0.54	-0.19
	H	-0.64	0.06
	C	0.01	-0.12
	C	-0.01	-0.20
	C	0.01	-0.19
	C	0.00	-0.25
	C	0.00	-0.24
	C	-0.01	-0.23
	H	0.02	0.25
	H	0.07	0.27
	H	0.01	0.25
	H	0.01	0.25

	H	0.01	0.24
$[B_{12}H_{10}OHNH_3]^-$			
	O	-1.40	-0.97
	N	-1.32	-0.94
	H	0.45	0.45
	$B_{O\text{-sub.}}$	0.63	0.37
	$B_{N\text{-sub.}}$	0.01	0.09
	B	0.51	-0.23
	H	-0.65	0.04
	B	0.54	-0.21
	H	-0.64	0.05
	B	0.57	-0.19
	H	-0.64	0.06
	B	0.53	-0.22
	H	-0.64	0.05
	B	0.57	-0.19
	H	-0.65	0.05
	B	0.57	-0.19
	H	-0.65	0.05
	B	0.57	-0.17
	H	-0.64	0.05
	B	0.55	-0.18
	H	-0.64	0.05
	B	0.57	-0.17
	H	-0.64	0.05
	B	0.55	-0.18
	H	-0.64	0.05
	H	0.59	0.51
	H	0.45	0.45

	H	0.50	0.47

Table S3. NBO и QTAIM atomic charges of $[B_nH_{n-2}ONHCR]^-$ and $[B_nH_{n-2}OHNH_3]^-$ (n = 10, 12; R = H, CH_3, C_3H_7, *iso*-C_3H_7, C_6H_5). $C_{carb.}$ – carbonyl C-atom of diboraoxazole rings. $B_{O\text{-}sub.}$ – boron atom with attached O-atom. $B_{N\text{-}sub.}$ – boron atom with attached N-atom.

Table S4. Cartesian atomic coordinates of the calculated optimized equilibrium model structures. All coordinates are given in Angstrom units.

Compound	Atom	x	y	z
$[B_{10}H_8ONHCH]^-$				
	O	4.167487	8.725381	2.738678
	N	4.852468	9.853598	0.930389
	H	4.678768	10.164175	-0.01472
	C	3.951318	9.122949	1.536231
	B	5.518369	9.256105	3.158723
	B	6.075501	10.114164	1.828892
	B	5.845409	10.882777	3.451292
	H	4.953578	11.589946	3.840277
	B	6.77701	9.467299	4.22464
	H	6.829387	9.022637	5.33794
	B	6.984635	8.715995	2.531227
	H	7.064727	7.581948	2.138561
	B	7.827847	10.033797	1.565976
	H	8.252787	9.740132	0.478686
	B	7.023029	11.56498	2.21619
	H	6.740267	12.614237	1.698925
	B	7.54562	11.115331	3.924618
	H	7.784969	11.799351	4.883791
	B	8.350374	9.581439	3.270988
	H	9.287628	8.940519	3.665366
	B	8.618389	11.129273	2.591144
	H	9.648918	11.724409	2.463672
	H	3.014266	8.83046	1.064754
$[B_{10}H_8ONHCCH_3]^-$				
	O	4.147563	8.788011	2.715306
	N	4.864361	9.906646	0.923123

	H	4.713054	10.231134	-0.02071
	C	3.931942	9.189948	1.507772
	C	2.637768	8.803005	0.867437
	H	2.578656	7.713022	0.806468
	H	2.541212	9.226199	-0.13405
	B	5.502578	9.286184	3.147995
	B	6.084755	10.136638	1.827861
	B	5.863373	10.903553	3.452491
	H	4.98499	11.628651	3.840724
	B	6.75887	9.465111	4.223663
	H	6.794927	9.014324	5.335506
	B	6.961489	8.717552	2.529022
	H	7.020304	7.583093	2.132098
	B	7.837235	10.020717	1.574397
	H	8.262037	9.72275	0.487836
	B	7.061572	11.565619	2.226855
	H	6.80479	12.623322	1.712416
	B	7.565127	11.097749	3.936186
	H	7.814514	11.772426	4.899735
	B	8.341185	9.549793	3.280161
	H	9.262999	8.887922	3.677203
	B	8.645383	11.094668	2.608461
	H	9.689029	11.668823	2.489293
	H	1.809831	9.150126	1.489686
$[B_{10}H_8ONHCC_3H_7]^-$				
	O	4.148475	8.783564	2.723209
	N	4.859301	9.901313	0.928733
	H	4.702396	10.227764	-0.01358
	C	3.927509	9.185503	1.515713

	C	2.62261	8.809954	0.881149
	H	2.602207	7.716889	0.789108
	H	2.578394	9.223552	-0.1325
	C	1.420776	9.281639	1.708785
	H	1.466243	10.371784	1.817439
	H	1.506949	8.866794	2.717607
	C	0.092881	8.871534	1.075052
	H	-0.75417	9.209242	1.680147
	H	-0.02199	9.300959	0.07307
	H	0.02019	7.781883	0.979789
	B	5.504002	9.281857	3.151499
	B	6.082262	10.131912	1.829198
	B	5.862331	10.900048	3.453452
	H	4.981924	11.623291	3.841363
	B	6.761433	9.464353	4.224881
	H	6.800576	9.014791	5.33715
	B	6.962466	8.715097	2.530346
	H	7.023503	7.580267	2.135012
	B	7.834383	10.019306	1.572977
	H	8.258083	9.72114	0.486132
	B	7.057188	11.563229	2.225316
	H	6.79705	12.619799	1.710197
$[B_{10}H_8ONHC$*iso*-$C_3H_7]^-$				
	O	4.545119	15.094204	7.261074
	N	5.524047	15.652506	9.185458
	H	5.804706	16.362509	9.846274
	C	4.897073	16.019009	8.091194
	C	4.564064	17.44433	7.738482
	H	4.918278	18.076719	8.562527

	C	5.296365	17.845393	6.451487
	H	6.379777	17.733214	6.558408
	H	4.973579	17.212886	5.619865
	H	5.077381	18.8891	6.20235
	C	3.045027	17.606514	7.602393
	H	2.529961	17.324035	8.525598
	H	2.795549	18.647163	7.369828
	H	2.667643	16.968574	6.798284
	B	4.979504	13.76403	7.815978
	B	5.724721	14.131428	9.2696
	B	6.584597	13.266872	7.931546
	H	7.378608	13.691274	7.132293
	B	5.132816	12.107734	7.787579
	H	4.737143	11.38086	6.918133
	B	4.291393	13.032335	9.168103
	H	3.135983	13.258043	9.421395
	B	5.491819	12.934593	10.55747
	H	5.121219	13.291779	11.64609
	B	7.111387	13.100144	9.683378
	H	8.155188	13.608238	10.00317
	B	6.699813	11.639183	8.639799
	H	7.40135	10.826574	8.098708
	B	5.075965	11.474217	9.517183
	H	4.384169	10.515976	9.736432
	B	6.564425	11.637781	10.34487
	H	7.062992	10.893172	11.13874
$[B_{10}H_8ONHCC_6H_5]^-$				
	O	12.432294	2.108993	0.036448
	N	10.198133	2.288306	-0.00743

	H	9.290019	1.86241	0.098086
	C	11.274412	1.538757	0.07075
	B	12.267497	3.619568	-0.09148
	B	10.552264	3.781635	-0.12982
	B	11.445156	4.33821	1.297013
	H	11.359588	3.696354	2.310609
	B	12.99132	5.004194	0.70995
	H	13.995891	4.935388	1.361157
	B	13.015164	4.854369	-1.08993
	H	14.034761	4.682421	-1.69591
	B	11.483943	4.09839	-1.60169
	H	11.428054	3.297598	-2.49782
	B	10.102812	5.13562	-1.15456
	H	9.093081	5.163284	-1.8033
	B	10.078634	5.282723	0.647804
	H	9.053673	5.415377	1.257834
	B	11.614843	6.074308	1.148161
	H	11.659809	6.786592	2.112039
	B	12.57789	6.401195	-0.31769
	H	13.295451	7.359362	-0.38742
	B	11.654337	5.836276	-1.73541
	H	11.729896	6.3787	-2.80172
	B	10.791723	6.570491	-0.35643
	H	10.271334	7.645927	-0.45114
	C	11.24204	0.064659	0.214158
	C	10.16466	-0.689469	-0.25707
	C	12.321432	-0.574443	0.829116
	C	10.16055	-2.072037	-0.10041
	C	12.311634	-1.955811	0.98824

	C	11.231265	-2.706352	0.526593
	H	9.341776	-0.203702	-0.77467
	H	13.156313	0.026478	1.174657
	H	9.32476	-2.654606	-0.47691
	H	13.149746	-2.448134	1.47303
	H	11.227016	-3.78622	0.648244
$[B_{10}H_8OHNH_3]^-$				
	O	4.265467	8.456444	3.248002
	N	4.956435	9.933854	0.823155
	H	5.348673	9.561813	-0.03818
	B	5.546594	9.143035	3.292097
	B	6.057999	10.033312	1.95269
	B	5.85713	10.79115	3.56337
	H	4.981935	11.489063	4.00363
	B	6.889074	9.440055	4.267498
	H	6.980592	8.978915	5.3743
	B	7.029126	8.67914	2.591289
	H	7.155571	7.545687	2.198707
	B	7.792807	10.026005	1.598018
	H	8.142274	9.746793	0.474804
	B	6.963309	11.513364	2.281515
	H	6.597587	12.537594	1.754444
	B	7.592186	11.098333	3.953006
	H	7.840519	11.7923	4.902842
	B	8.414175	9.598688	3.266284
	H	9.387196	8.985473	3.616932
	B	8.597742	11.153444	2.573241
	H	9.59057	11.793823	2.37852
	H	4.235262	9.299298	1.189721

	H	4.550377	10.847015	0.635355
	H	4.393777	7.531297	3.463246
$[B_{12}H_{10}ONCH]^-$				
	O	12.51453	2.15546	0.053024
	N	10.27006	2.281255	0.022032
	H	9.378889	1.80967	0.059369
	C	11.369315	1.576709	0.096284
	B	12.30688	3.667434	-0.08709
	B	10.585088	3.786661	-0.11325
	B	11.474967	4.377461	1.300571
	H	11.412789	3.743537	2.320526
	B	12.999642	5.077882	0.696218
	H	14.00979	5.041635	1.340636
	B	13.014752	4.912838	-1.10214
	H	14.034018	4.760506	-1.71388
	B	11.499915	4.112309	-1.5954
	H	11.457145	3.302281	-2.484
	B	10.095264	5.117718	-1.14775
	H	9.080817	5.111495	-1.78958
	B	10.079894	5.281889	0.653571
	H	9.056335	5.394208	1.269905
	B	11.599204	6.116048	1.134778
	H	11.63299	6.838634	2.091228
	B	12.542558	6.454362	-0.34103
	H	13.233558	7.430492	-0.42482
	B	11.624249	5.852678	-1.74687
	H	11.677624	6.386513	-2.81874
	B	10.752881	6.577404	-0.36808
	H	10.204678	7.638333	-0.4688

	H	11.356982	0.492651	0.195972
$[B_{12}H_{10}ONCCH_3]^-$				
	O	12.576981	2.172798	0.086745
	N	10.337902	2.243279	0.058067
	H	9.45327	1.761085	0.101141
	C	11.448685	1.549248	0.141143
	B	12.33716	3.671369	-0.0716
	B	10.615522	3.750442	-0.0968
	B	11.491443	4.381206	1.307907
	H	11.445657	3.758461	2.336229
	B	12.998525	5.107466	0.693451
	H	14.010116	5.103533	1.337006
	B	13.016062	4.920732	-1.10272
	H	14.038665	4.784118	-1.71312
	B	11.520144	4.080945	-1.58417
	H	11.495543	3.258637	-2.46303
	B	10.093207	5.057176	-1.14766
	H	9.078336	5.019326	-1.78791
	B	10.075557	5.243379	0.651798
	H	9.049538	5.340622	1.266914
	B	11.575443	6.119306	1.120711
	H	11.593737	6.856851	2.066322
	B	12.50975	6.460769	-0.36048
	H	13.176753	7.452263	-0.45712
	B	11.60397	5.821286	-1.75807
	H	11.643968	6.345049	-2.83562
	B	10.717236	6.542139	-0.38684
	H	10.145483	7.589446	-0.4999
	C	11.495079	0.063451	0.293124

	H	10.495547	-0.367882	0.371256
	H	12.010367	-0.367097	-0.56935
	H	12.070681	-0.1852	1.187942
$[B_{12}H_{10}ONCC_3H_7]^-$				
	O	12.515851	2.162251	0.051103
	N	10.279474	2.281177	0.022459
	H	9.381547	1.824236	0.062387
	C	11.373601	1.561142	0.098587
	B	12.309379	3.666777	-0.08826
	B	10.591294	3.783199	-0.11367
	B	11.477793	4.377968	1.299512
	H	11.416843	3.743243	2.320089
	B	13.000527	5.079239	0.695792
	H	14.011077	5.045227	1.340379
	B	13.015872	4.915009	-1.10257
	H	14.035859	4.765245	-1.71447
	B	11.503006	4.113897	-1.59605
	H	11.461679	3.303392	-2.48547
	B	10.096815	5.114303	-1.14787
	H	9.08188	5.106602	-1.78952
	B	10.081264	5.277813	0.653237
	H	9.056946	5.388189	1.269391
	B	11.598511	6.116173	1.134781
	H	11.630939	6.840089	2.090682
	B	12.541603	6.456028	-0.3409
	H	13.230769	7.433829	-0.42425
	B	11.623943	5.853942	-1.74719
	H	11.676389	6.39	-2.81835
	B	10.750998	6.57586	-0.36794

	H	10.200737	7.636092	-0.46818
	C	11.404819	0.067363	0.236771
	H	11.956933	-0.315828	-0.63009
	H	12.027509	-0.152458	1.11197
	C	10.046277	-0.619038	0.35757
	H	9.509004	-0.219362	1.227043
	H	9.437742	-0.393109	-0.52761
	C	10.18368	-2.13457	0.499754
	H	10.770145	-2.395194	1.387705
	H	10.687632	-2.568364	-0.37103
	H	9.20475	-2.614007	0.593556
$[B_{12}H_{10}ONC$*iso*-$C_3H_7]^-$				
	O	12.556327	2.171005	0.151945
	N	10.322738	2.253333	0.020298
	H	9.432167	1.780568	0.01298
	C	11.422923	1.550051	0.147345
	B	12.335128	3.668124	-0.02227
	B	10.617395	3.758072	-0.12503
	B	11.431783	4.393608	1.312263
	H	11.335907	3.77962	2.34229
	B	12.96965	5.106213	0.760976
	H	13.949739	5.101822	1.452204
	B	13.067238	4.906752	-1.03095
	H	14.114615	4.759812	-1.59552
	B	11.58983	4.072397	-1.57221
	H	11.597642	3.241637	-2.44451
	B	10.150366	5.059565	-1.20818
	H	9.166505	5.022644	-1.89585
	B	10.052203	5.259681	0.587056

	H	9.000781	5.366981	1.155849
	B	11.534905	6.129767	1.116217
	H	11.515967	6.873303	2.057401
	B	12.537509	6.454737	-0.32314
	H	13.214903	7.441613	-0.39621
	B	11.691775	5.81038	-1.75476
	H	11.78344	6.32491	-2.83371
	B	10.748283	6.546669	-0.43099
	H	10.187913	7.595971	-0.57798
	C	11.47766	0.047426	0.244078
	H	12.162126	-0.161471	1.075269
	C	10.123102	-0.594556	0.540509
	H	9.419751	-0.45287	-0.28927
	H	10.246232	-1.673387	0.676155
	H	9.67329	-0.192264	1.453733
	C	12.103821	-0.504489	-1.04671
	H	13.084177	-0.056978	-1.22321
	H	12.219075	-1.59114	-0.97606
	H	11.46886	-0.278145	-1.91002
$[B_{12}H_{10}ONCC_6H_5]^-$				
	O	12.432294	2.108993	0.036448
	N	10.198133	2.288306	-0.00743
	H	9.290019	1.86241	0.098086
	C	11.274412	1.538757	0.07075
	B	12.267497	3.619568	-0.09148
	B	10.552264	3.781635	-0.12982
	B	11.445156	4.33821	1.297013
	H	11.359588	3.696354	2.310609
	B	12.99132	5.004194	0.70995

	H	13.995891	4.935388	1.361157
	B	13.015164	4.854369	-1.08993
	H	14.034761	4.682421	-1.69591
	B	11.483943	4.09839	-1.60169
	H	11.428054	3.297598	-2.49782
	B	10.102812	5.13562	-1.15456
	H	9.093081	5.163284	-1.8033
	B	10.078634	5.282723	0.647804
	H	9.053673	5.415377	1.257834
	B	11.614843	6.074308	1.148161
	H	11.659809	6.786592	2.112039
	B	12.57789	6.401195	-0.31769
	H	13.295451	7.359362	-0.38742
	B	11.654337	5.836276	-1.73541
	H	11.729896	6.3787	-2.80172
	B	10.791723	6.570491	-0.35643
	H	10.271334	7.645927	-0.45114
	C	11.24204	0.064659	0.214158
	C	10.16466	-0.689469	-0.25707
	C	12.321432	-0.574443	0.829116
	C	10.16055	-2.072037	-0.10041
	C	12.311634	-1.955811	0.98824
	C	11.231265	-2.706352	0.526593
	H	9.341776	-0.203702	-0.77467
	H	13.156313	0.026478	1.174657
	H	9.32476	-2.654606	-0.47691
	H	13.149746	-2.448134	1.47303
	H	11.227016	-3.78622	0.648244
$[B_{12}H_{10}OHNH_3]^-$				

	O	12.356345	2.119924	0.009247
	N	9.485654	2.442282	-0.00423
	H	8.882579	2.421051	0.814032
	B	11.989073	3.528541	-0.10667
	B	10.261743	3.810703	-0.12627
	B	11.163998	4.31141	1.281895
	H	11.057409	3.690062	2.31156
	B	12.709717	4.945701	0.672567
	H	13.721512	4.849154	1.315839
	B	12.713169	4.800725	-1.11333
	H	13.724451	4.606314	-1.72825
	B	11.172323	4.060445	-1.60091
	H	11.061094	3.282911	-2.51543
	B	9.832687	5.162089	-1.1435
	H	8.805383	5.183642	-1.77104
	B	9.829932	5.319401	0.646846
	H	8.802076	5.445968	1.260509
	B	11.379114	6.056199	1.137513
	H	11.457575	6.7658	2.102059
	B	12.330831	6.361013	-0.34331
	H	13.078064	7.296596	-0.419
	B	11.380657	5.812654	-1.74914
	H	11.454547	6.348194	-2.81958
	B	10.558333	6.587357	-0.36287
	H	10.060704	7.6741	-0.45618
	H	13.001229	2.016293	0.711175
	H	8.942366	2.238552	-0.83938
	H	10.236186	1.743729	0.097766
